# *EGFR*-activating mutations, DNA copy number abundance of ErbB family, and prognosis in lung adenocarcinoma

**DOI:** 10.18632/oncotarget.7029

**Published:** 2016-01-27

**Authors:** Hsuan-Yu Chen, Chia-Hsin Liu, Ya-Hsuan Chang, Sung-Liang Yu, Bing-Ching Ho, Chung-Ping Hsu, Tsung-Ying Yang, Kun-Chieh Chen, Kuo-Hsuan Hsu, Jeng-Sen Tseng, Jiun-Yi Hsia, Cheng-Yen Chuang, Chi-Sheng Chang, Yu-Cheng Li, Ker-Chau Li, Gee-Chen Chang, Pan-Chyr Yang

**Affiliations:** ^1^ Institute of Statistical Sciences, Academia Sinica, Taipei, Taiwan; ^2^ College of Medicine, National Taiwan University, Taipei, Taiwan; ^3^ College of Life Science, National Taiwan University, Taipei, Taiwan; ^4^ Bioinformatics Program, Taiwan International Graduate Program, Academia Sinica, Taipei, Taiwan; ^5^ Institute of Biomedical Informatics, National Yang-Ming University, Taipei, Taiwan; ^6^ Center of Genomic Medicine, National Taiwan University, Taipei, Taiwan; ^7^ Taichung Veterans General Hospital, Taichung, Taiwan; ^8^ Faculty of Medicine, School of Medicine, National Yang-Ming University, Taipei, Taiwan; ^9^ Comprehensive Cancer Center, Taichung Veterans General Hospital, Taichung, Taiwan; ^10^ Department of Internal Medicine, National Taiwan University Hospital, Taipei, Taiwan; ^11^ Institute of Biomedical Sciences, Academia Sinica, Taipei, Taiwan

**Keywords:** lung adenocarcinoma, DNA copy number abundance, ErbB family, EGFR-activating mutation, prognosis

## Abstract

In this study, *EGFR*-activating mutation status and DNA copy number abundances of members of ErbB family were measured in 261 lung adenocarcinomas. The associations between DNA copy number abundances of ErbB family, *EGFR*-activating mutation status, and prognosis were explored. Results showed that DNA copy number abundances of *EGFR*, *ERBB2*, *ERBB3*, and *ERBB4* had associations with overall survival in lung adenocarcinoma with *EGFR*-activating mutations. In the stratification analysis, only *ERBB2* showed significant discrepancy in patients carrying wild type *EGFR* and other members of ErbB family in patients carrying *EGFR*-activating mutation. This indicated that CNAs of ErbB family had effect modifications of *EGFR*-activating mutation status. Findings of this study demonstrate potential molecular guidance of patient management of lung adenocarcinoma with or without *EGFR*-activating mutations.

## INTRODUCTION

Lung cancer was the leading cause of cancer death worldwide [1]. It was divided into small-cell lung cancer (SCLC, comprising 20% of lung cancers), and non-small-cell lung cancer (NSCLC, comprising 80% of lung cancers). The global 5-year survival rates of NSCLC remained low, ranging from 10% to 15%[1]. NSCLC is characterized by the accumulation of multiple genetic alterations that results from the inactivation of tumor suppressor genes, activation of oncogenes, and epigenetic changes. Among all subtypes of NSCLC, adenocarcinoma was the most common type found in women and in non-smokers [2, 3]. Currently, surgery remains the gold standard for early stage NSCLC.

The receptor tyrosine kinase (RTK) super-family of cell surface receptors serves as mediators of cell signaling by extra-cellular growth factors [4–6]. ErbB family was one of RTK super-family. The ErbB family including *EGFR* (also known as *ERBB1* or *HER1*), *ERBB2* (also known as *HER2*), *ERBB3* (also known as *HER3*), and *ERBB4* (also known as *HER4*) had received much attention and their strong association with malignant proliferation had been investigated [6–9]. Activation of ErbB family proteins stimulates many intracellular signaling pathways such as MAPK and (PI3K)–AKT pathways [10, 11]. Other important ErbB signaling effectors are the signal transducer and activator of transcription proteins such as STATs [12], which often associates with *EGFR* activation [13], SRC tyrosine kinase, the activity of which is increased in response to *EGFR* and *ERBB2* signalling [14], and mammalian target of rapamycin (mTOR), a serine/threonine kinase activates downstream of I3K—AKT and other growth regulators [15].

In this study, *EGFR*-activating mutation status and DNA copy number abundances of *EGFR*, *ERBB2*, *ERBB3*, and *ERBB4* were measured in 261 surgically resected lung adenocarcinomas. In addition, the associations between DNA copy number abundances of above genes, *EGFR*-activating mutation status, and prognosis were explored. The findings of this study may provide potential biomarkers for drug response and prognosis of lung adenocarcinoma.

*EGFR* genomic DNA amplification leading to mRNA overexpression was often found in various types of human cancer [16, 17]. Increased mRNA expression levels of *EGFR* were observed in various cancers such as head and neck, ovary, cervix, bladder, oesophagus, stomach, brain, breast, endometrium, colon and lung, and frequently conferred an adverse prognosis [4, 18].

Extending previous observations of almost two decades ago [19, 20], recent retrospective analyses had reported *EGFR* overexpression in 62% of NSCLC cases, and its expression was correlated with DNA copy number abundance and poor prognosis [18, 21, 22]. Although DNA copy number abundance of *EGFR* and *ERBB2* had been studied independently [23, 24], the associations between *EGFR*-activating mutations, whole ErbB family, and clinical prognosis in lung cancer were still needed to be investigated.

## RESULTS

### Clinical characteristics of patients

Among 261 patients, there were 163 patients (66.3%) with stage I disease, 33 (12.6%) with stage II, and 65 (24.9%) with stage IIIA, respectively. There were 70 patients with *EGFR* L858R mutation (26.8%), 73 patients with *EGFR* exon-19-deletion (28.0%), and 118 *EGFR* wild type patients (45.2%), respectively (Table 1). There were 131 male (50.2%) and 130 female (49.8%). Many male (63.36 %) were current or ex-smoker and only 3 female (2.31 %) were smoker. The percentage of female had *EGFR*-activating mutation (63.08%) was higher than male (46.56%) (p-value = 0.009, Fisher's exact test). Never smokers had higher *EGFR*-activating mutations (63.31%) comparing to smokers or ex-smokers (39.53%) (p-value = 0.0005, Fisher's exact test). Patients with *EGFR*-activating mutation had higher *EGFR* CNAs than patients without *EGFR*-activating mutation (p-value = 0.009, student t-test).

**Table 1:** Clinical characteristics of patients

Variable	All (%)	Wild type (%)	L858R (%)	Del-19 (%)
Total patients	261	118	70	73
Gender				
Male	131 (50.2)	70 (59.32)	24 (34.28)	37 (50.69)
Female	130 (49.8)	48 (40.67)	46 (65.72)	36 (49.31)
Smoking status				
Non-smoker	169 (66.3)	62 (54.38)	53 (75.71)	54 (76.05)
Ex-smoker	40 (15.7)	23 (20.17)	8 (11.42)	9 (12.67)
Current smoker	46 (18.0)	29 (25.43)	9 (12.85)	8 (11.26)
Smoking years				
0	169 (66.3)	62 (54.38)	53 (75.71)	54 (76.05)
≤20	23 (9.0)	11 (9.64)	7 (10.00)	5 (7.04)
21-40	41 (16.1)	25 (21.92)	7 (10.00)	9 (12.67)
>40	22 (8.6)	16 (14.03)	3 (4.28)	3 (4.22)
Dose of cigarette smoking				
0 package	169 (66.3)	62 (54.38)	53 (75.71)	54 (76.05)
≤20 packages	28 (11.0)	10 (8.77)	9 (12.85)	9 (12.67)
21-40 packages	26 (10.2)	18 (13.15)	5 (7.14)	3 (4.22)
>40 packages	32(12.6)	24 (21.05)	3 (4.28)	5 (7.04)
Smoking-quitted for year				
Non-smoker	169 (66.3)	62 (54.38)	53 (75.71)	54 (76.05)
Quitted > 15 years	14 (5.5)	8 (7.01)	3 (4.28)	3 (4.22)
Quitted < 15 years	26 (10.2)	15 (13.15)	5 (7.14)	6 (8.45)
Current smoker	46 (18.0)	29 (25.43)	9 (12.85)	8 (11.26)
Histology type				
Adenocarcinoma	249 (95.4)	110 (93.22)	68 (97.14)	71 (97.26)
BAC	12 (4.6)	8 (6.77)	2 (2.85)	2 (2.73)
Stage				
I	163 (62.5)	67 (56.77)	47 (67.14)	49 (67.12)
II	33 (12.6)	21 (17.79)	8 (11.42)	4 (5.47)
IIIA	65 (24.9)	30 (25.42)	15 (21.42)	20 (27.39)
Mutation status				
Wild type	118 (45.2)	118 (100)		
L858R	70 (26.8)		70(100)	
Exon-19-deletion	73 (28.0)			73(100)

### Patients with higher DNA copy number abundance of ErbB family had shorten overall survival

**Figure 1:**
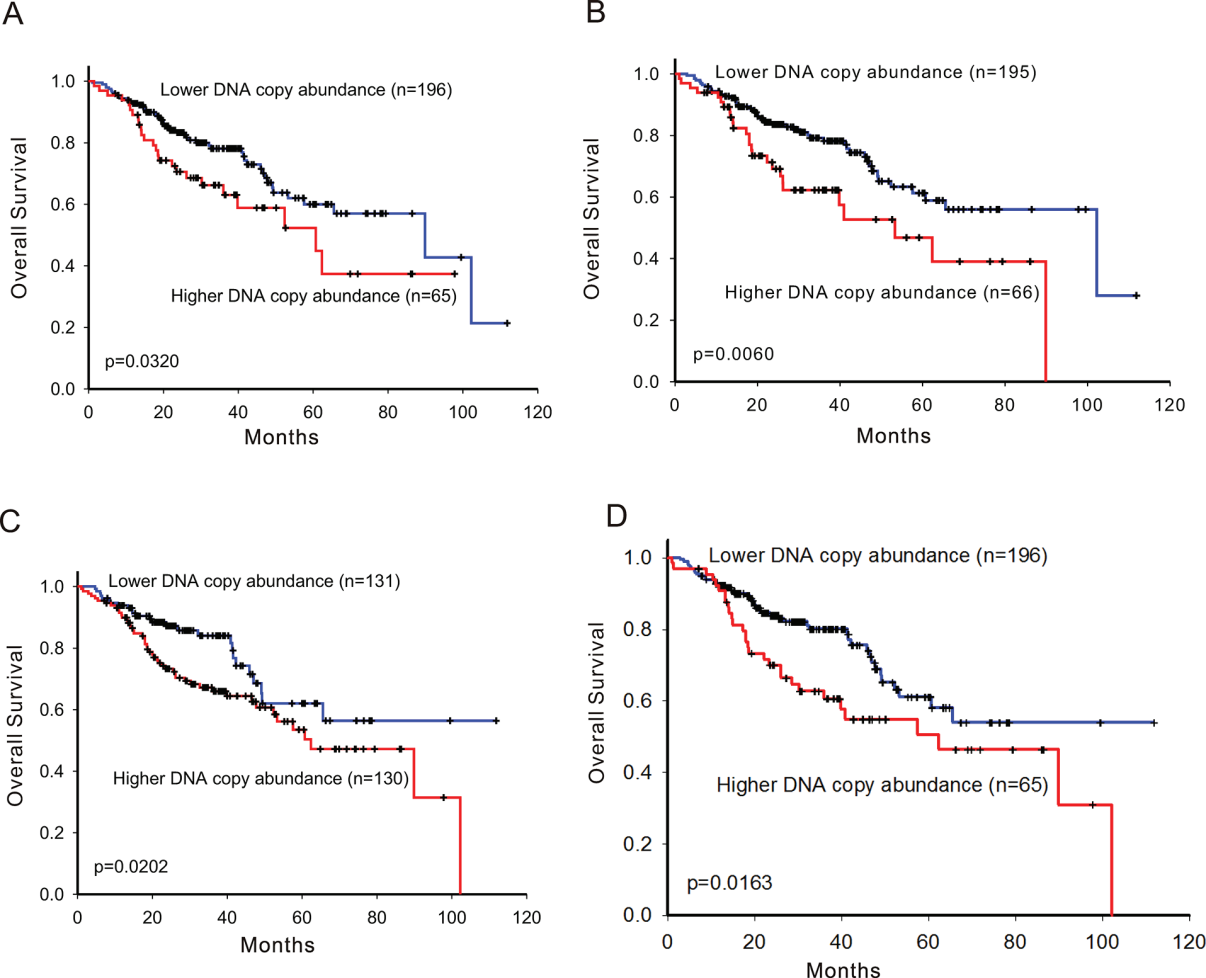
Survival prediction by DNA copy number abundance of ErbB family in 261 patients Kaplan-Meier curves for overall survival analysis on **A.** *EGFR*, **B.** *ERBB2*, **C.** *ERBB3*, and **D.** *ERBB4*. High- and low-risk groups are divided based on copy number abundance. The optimal cut points were determined by sensitivity analysis which provided the largest discrepancy in overall survival between the low- and high-risk groups on the basis of the log-rank statistic, respectively.

The results of sensitivity analysis showed that 75% percentile of *EGFR, ERBB2, ERBB4* and 50% percentile of *ERBB3* CNA were the optimal cut-off points for group separation, respectively (Supplementary Tables S1, S2, S3, and S4). Patients with high ErbB family CNAs significantly had shorten overall survival than patients with lower CNAs (Figure 1). Multivariate Cox proportional hazards regression analysis with other clinical covariates adjustments showed that CNAs of ErbB family were all significant prognostic factors (Table 2).

**Table 2:** Results of multivariate Cox regression

Gene	Adjusted HR^$^	95% C.I.		P value
	**All patients (*n* = 261)**			
*EGFR*	1.89	1.16	3.10	0.011
*ERBB2*	1.68	1.03	2.74	0.038
*ERBB3*	1.65	1.02	2.68	0.042
*ERBB4*	1.62	1.01	2.61	0.047
	**Wild type (*n* = 118)**			
*EGFR*	1.13	0.58	2.22	0.718
*ERBB2*	1.84	0.96	3.55	0.068
*ERBB3*	0.94	0.51	1.73	0.841
*ERBB4*	1.23	0.64	2.37	0.527
	**EGFR-activating mutations (*n* = 143)**			
*EGFR*	3.53	1.58	7.87	0.002
*ERBB2*	2.00	0.91	4.40	0.086
*ERBB3*	2.64	1.17	5.95	0.019
*ERBB4*	3.40	1.55	7.48	0.002
	**L858R (*n* = 70)**			
*EGFR*	2.96	1.02	8.57	0.046
*ERBB2*	2.86	0.91	9.03	0.074
*ERBB3*	3.98	1.02	15.63	0.047
*ERBB4*	7.22	2.23	23.36	0.001
	**Exon 19 deletion (*n* = 73)**			
*EGFR*	7.25	1.74	30.27	0.007
*ERBB2*	2.47	0.64	9.60	0.192
*ERBB3*	3.39	0.88	13.03	0.076
*ERBB4*	1.88	0.53	6.71	0.332

$ Hazard ratio

+ *EGFR*-activating mutations include L858R and exon-19-deletion

The association between members of ErbB family DNA copy number abundance and overall survival was evaluated by multivariate Cox hazard regression in all 261 patients, 118 wild type patients, 70 *EGFR* L858R patients, 73 *EGFR* exon-19-deletion patients, and all 143 *EGFR*-activating mutation patients. Potential confounding factors such age, gender, stage, and cell type were adjusted. Hazard ratio, confidence interval, and p-value were shown.

### Patients carrying wild type *EGFR* with higher DNA copy number abundance of *ERBB2* had shorten overall survival

**Figure 2:**
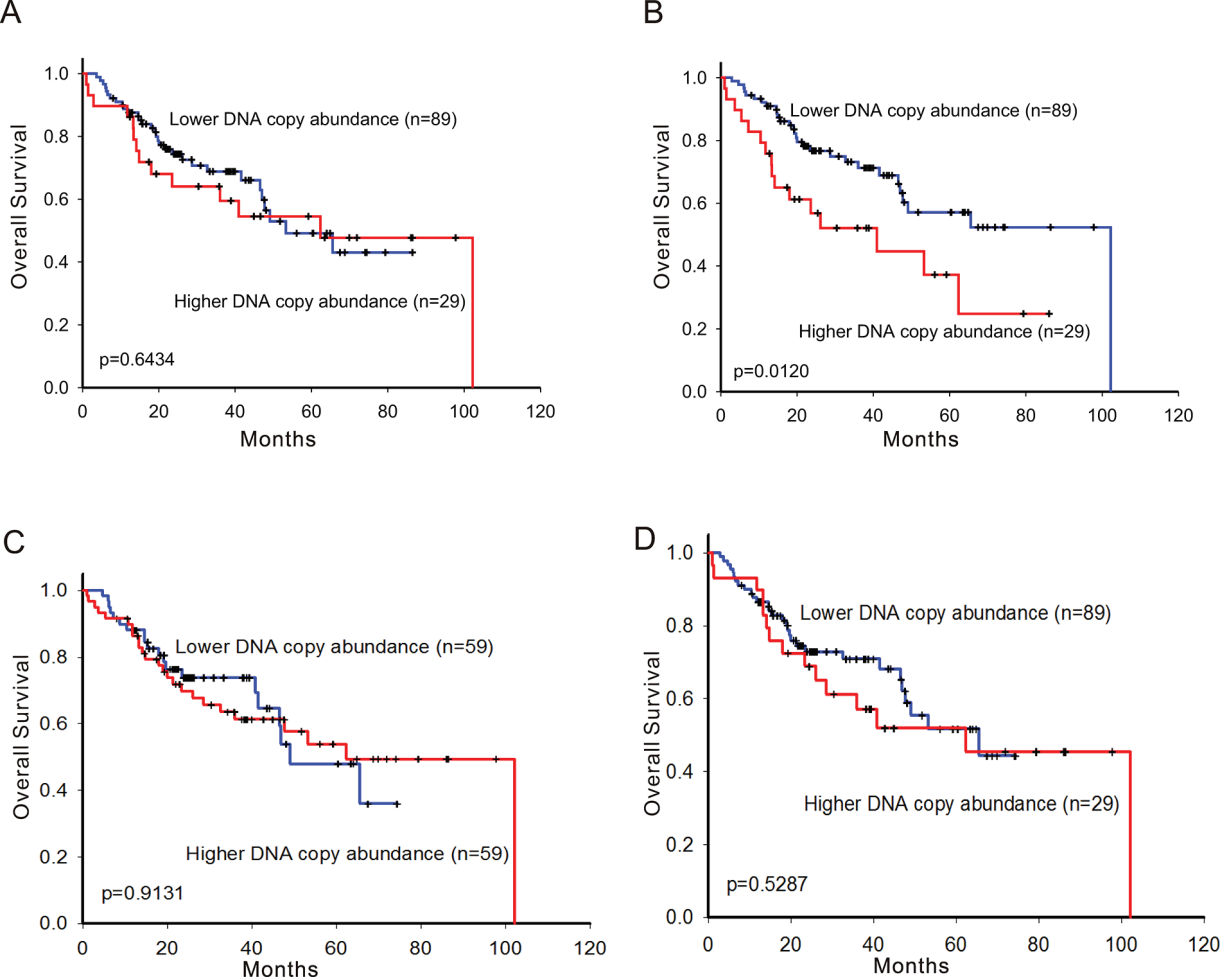
Survival prediction by DNA copy number abundance of ErbB family in 118 patients carrying wild type *EGFR* Kaplan-Meier curves for overall survival analysis on **A.** *EGFR*, **B.** *ERBB2*, **C.** *ERBB3*, and **D.** *ERBB4*. High- and low-risk groups are divided based on copy number abundance. The optimal cut points were determined by sensitivity analysis which provided the largest discrepancy in overall survival between the low- and high-risk groups on the basis of the log-rank statistic, respectively.

We further investigated the associations between CNAs of ErbB family and overall survival in different *EGFR*-activating mutation types of patients. In the stratification analysis, 143 patients were grouped into *EGFR*-activating mutation carrier group (70 in L858R and 73 in exon-19-deletion) and 118 patients were grouped into *EGFR* wild type carrier group, respectively. In the wild type *EGFR* group, patients with higher CNA of *ERBB2* significantly had shorten overall survival (Figure 2B) while those of *EGFR, ERBB3*, and *ERBB4* did not (Figure 2A, 2B, and 2D). Multivariate Cox proportional hazards regression analysis also showed that *ERBB2* was the only marginally significant prognostic factor in the wild type *EGFR* group (Table 2). This also implied that without driver mutations, such as *EGFR*-activating mutations, CNA of *ERBB2* outperformed CNAs of other ErbB family as a prognostic factor.

### Patients carrying *EGFR*-activating mutations with higher DNA copy number abundance of *EGFR, ERBB3,* and *ERBB4* had shorten overall survival

**Figure 3:**
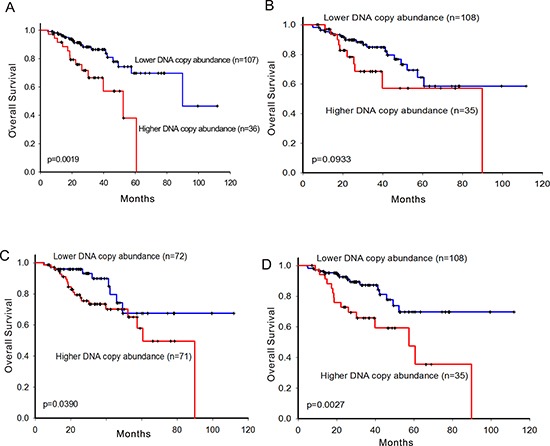
Survival prediction by DNA copy number abundance of ErbB family in 143 patients carrying *EGFR*-activating mutation Kaplan-Meier curves for overall survival analysis on **A.** *EGFR*, **B.** *ERBB2*, **C.** *ERBB3*, and **D.** *ERBB4*. High- and low-risk groups are divided based on copy number abundance. The optimal cut points were determined by sensitivity analysis which provided the largest discrepancy in overall survival between the low- and high-risk groups on the basis of the log-rank statistic, respectively.

In the *EGFR*-activating mutation carrier group, a different patterns was shown comparing with that in the wild type *EGFR* group. Patients with higher CNAs of *EGFR, ERBB3*, and *ERBB4* significantly had shorten overall survival (Figure 3A, 3C, and 3D) while that *ERBB2* did not (Figure 3B). Furthermore, *EGFR, ERBB3*, and *ERBB4* were significant prognostic factors evaluated by multivariate Cox proportional hazards regression analysis (Table 2). Comparing with results in the wild type *EGFR* group, this may imply that *ERBB2* showed different pattern with other members of ErbB family.

We further divided patientswith *EGFR*-activating mutations into L858R and exon 19 deletions two sub groups. There were 70 patients carrying L858R mutation and 73 patients carrying exon-19-deletion, respectively. Similar results were also found in the L858R group but not in the exon-19-deletion group (Supplementary Figure S1, Supplementary Figure S2, and Table 2). This may due to the complexity of exon-19-deletion which could be further categorized into many sub-types.

## DISCUSSION

The potential confounding factors were adjusted in the Cox proportional hazards regression analysis of testing association between CNAs and overall survival. In this study, the associations between CNAs and prognosis in lung adenocarcinoma were explored. All members of ErbB family showed significant associations between CNAs and overall survival of all patients. Furthermore, patients with higher CNAs of ErbB family showed poor prognosis. In the further stratified analysis of *EGFR*-activating mutation status, no significant association between CNAs and overall survival was found in the wild type group. It indicated that CNAs of ErbB family had effect modifications of different *EGFR*-activating mutation status. This may imply that without *EGFR*-activating mutation, CNAs of ErbB family may not affect the prognosis of patients except *ERBB2*.

Sensitivity analysis was used to explore the optimal CNAs cut-point of prognosis for each gene respectively. The optimal cut-points varied from 50% to 75% in different genes. It was because that CNA was a continuous variable and higher CNA was trend to poor prognosis. Hence, when patients were di-categorized into two groups, it may toward to result in higher cut-off point. Nevertheless, a specific cut-points could be found to discriminate patients into high-risk and low-risk groups significantly having different overall survival time and CNAs of ErbB family were still potentially biomarkers of prognosis in lung cancer.

In 2011, one study, an Asian cohort, showed that *EGFR* copy number gain *per se* had no significant associations with relapse-free survival and overall survival [25]. However, this findings may be due from only 34 patients had information on *EGFR* mutations and large-scale study was still needed. In this study, in term of relapse-free survival, patients with higher *EGFR* CNA had better relapse-free survival in *EGFR*-activating mutation group (HR= 1.77, 95% CI=1.00 to 3.12, p-value = 0.05) (Supplementary Table S5). However, other members of ErbB family had no association with relapse-free survival (Supplementary Table S5).

Although the basic structures of genes in the ErbB family are similar, each one has distinct properties, including variation in tyrosine kinase activity[26]. Except *ERBB2*, all other members of ErbB family showed significant associations between CNAs and prognosis in patients with *EGFR*-activating mutations. This may due to the biological role of *ERBB2*. *ERBB2* did not directly bind to any known ligand and functioned as a co-receptor binding tightly to other ligand-bound ErbB receptor family members. Such heterodimer may stabilize the ligand binding and may enhance kinase-mediated activation of downstream signaling pathways [6–9].

Among all members of ErbB family, only *ERBB2* CNA showed significant discrepancy between high-risk wild type patients and low-risk wild type patients. This may imply that without *EGFR*-activating mutations, the effect of *ERBB2* CNA, as a biomarker of prognosis, outperforms other members of ErbB family. In other studies, *ERBB2* had been reported as a significant biomarker of prognosis in other cancer types without *EGFR*-activating mutation such as breast cancer [27–29]. As a consequence, *ERBB2* CNA may be a valuable biomarker of prognosis in lung adenocarcinoma patients without *EGFR*-activating mutations.

CNAs of ErbB family showed effect modifications between different *EGFR*-activating mutation status. It indicated that CNAs of ErbB family predicted overall survival in patients with *EGFR*-activating mutations but not in wild type *EGFR*. Findings of this study demonstrated that CNAs of ErbB family provided prognosis prediction in patients with *EGFR*-activating mutations and provided potential molecular guidance of clinical management of lung adenocarcinoma. However, the prediction signature of patients with wild type *EGFR* is still not clear. It is necessary to collect more CNAs of cancer associated genes for investigation in the future.

## MATERIALS AND METHODS

All the lung adenocarcinoma patients underwent surgical resection from January 2001 to March 2009 in Taichung Veterans General Hospital. The lung tumor lesions were completely resected with lymph node dissection. Thepathological diagnoses were based on the 2004 World Health Organization histologic classification system [30]. TNM (tumor, node, and metastases) staging system was used according to the 6th edition of the American Joint Committee for Cancer (AJCC) staging system [31]. Only *EGFR* exon 19 deletion and L858R point mutation and *EGFR* wild type patients were included as other *EGFR* mutations were rare and heterogeneous. Patients with less than 3 months follow-up were excluded. This study was approved by the institutional review boards of Taichung Veterans General Hospital (TCVGH), with written informed consent from all patients.

### DNA extraction from frozen tumor tissue for genetic tests

The frozen lung cancer tissues were obtained at surgery, immediately snap frozen in liquid nitrogen and stored until use. Tumor specimens were procured for *EGFR* gene mutational analysis with previous description [32]. Briefly, DNA was extracted from the tumors using a QIAmp DNA Mini kit (Qiagen, Valencia, CA) following the manufacturer's protocols.

### Genotyping of EGFR mutation status

The identification of *EGFR*-activating mutation was genotyped by Matrix Assisted Laser Desorption Ionisation Time-of-Flight Mass Spectrometry (MALDI-TOF MS) or Sanger sequence assays[33]. The MALDI-TOF MS was performed by the MassARRAY system (SEQUENOM, San Diego, CA) followed standard protocol. In the biochemical reaction, polymerase chain reaction (PCR) followed by single nucleotide extension was performed by using primers and corresponding detection probes to amplify the region containing each target mutation. After SpectroClean Resin clean up, samples were loaded onto the matrix of SpectroCHIP® by Nanodispenser (Matrix) and then analyzed by Bruker Autoflex MALDI-TOF MS. Data were collected and analyzed by Typer 4 software (Sequenom, San Diego, CA).

### DNA copy number abundance

Due to the heterogeneity of cancer cells, the DNA copy number of cancer cells may not be measured identically in the tumor. It resulted that the copy number may not be an integer comparing with normal cells. Hence, we quantified DNA copy number abundance instead of categorizing CNA value into an integer such as 1, 2, or 3 copies.

The genomic real-time quantitative PCR (qPCR) was performed to measure DNA copy number abundance of each member of ErbB family. The primers and probes of qPCR were designed based on 500 franking nucleotide sequences (250 upstream and 250 downstream nucleotides) of the gene location. Fluorescence emitted by the reporter dye was detected in real time using the ABI prism 7900 sequence detection system (Applied Biosystem, Foster City, CA). Copy-number abundance (CNA) was defined as 2^−ΔCt^ which represents the copy number fold-change between the target gene and the internal control gene GAPDH.

### Statistical analysis

Clinical data collected including patient's age, gender, stage, smoking status (nonsmoking defined as patients had never smoked), date of diagnosis, progression, death or last follow-up. Overall survival (OS) was calculated from the date of surgery to the date of death. Patients were classified into the high or low risk groups based on CNAs of ErbB family. The sensitivity analysis was performed to select the optimal cut-off point of the best group separation for each gene, respectively. We screened every 5% from 20% to 75% of CNAs as cut points to evaluate the trend of multivariate Cox proportional hazards regression p-values and found that they gradually descended form 20% to the optimal cut-off point, which p-value was firstly smaller than 0.05, in each gene (Supplementary Tables S1, S2, S3, and S4).

The stratified analysis was performed to analyze the DNA CNAs in 4 groups (L858R, exon 19 deletion, activating mutations, and wild type) of different *EGFR* mutation status. The Kaplan-Meier method was used to estimate survival curves and the difference between survival curves was evaluated by the log-rank test. Multivariate Cox proportional hazards regression with covariates age, sex, and stage was used to evaluate independent prognostic factors associated with overall survival. All tests were two-tailed and p values less than 0.05 were considered to be significant.

## SUPPLEMENTARY FIGURES AND TABLES




