# Supplementary material for: EGFR-activating mutations, DNA copy number abundance of ErbB family, and prognosis in lung adenocarcinoma
Source: Oncotarget. 2016 Jan 27;7(8):9017–25. doi: 10.18632/oncotarget.7029 (PMC4891022; doi:10.18632/oncotarget.7029)
Supplement: Supplementary file 1 [file oncotarget-07-9017-s001.pdf]

SUPPLEMENTARY FIGURES AND TABLES

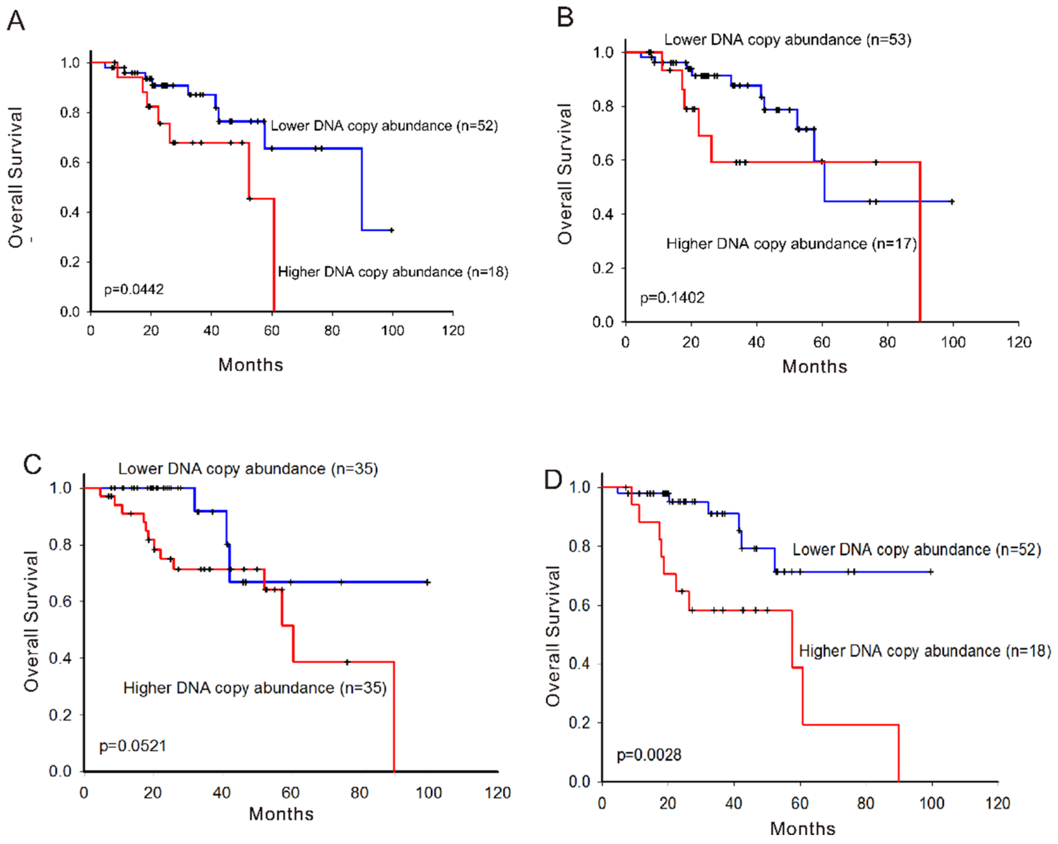

Supplementary Figure S1: Survival prediction by DNA copy number abundance of ErbB family in 70 patients carrying L858R mutation. Kaplan-Meier curves for overall survival analysis on **A.** *EGFR*, **B.** *ERBB2*, **C.** *ERBB3*, and **D.** *ERBB4*. High- and low-risk groups are divided based on copy number abundance. The optimal cut points were determined by sensitivity analysis which provided the largest discrepancy in overall survival between the low- and high-risk groups on the basis of the log-rank statistic, respectively.

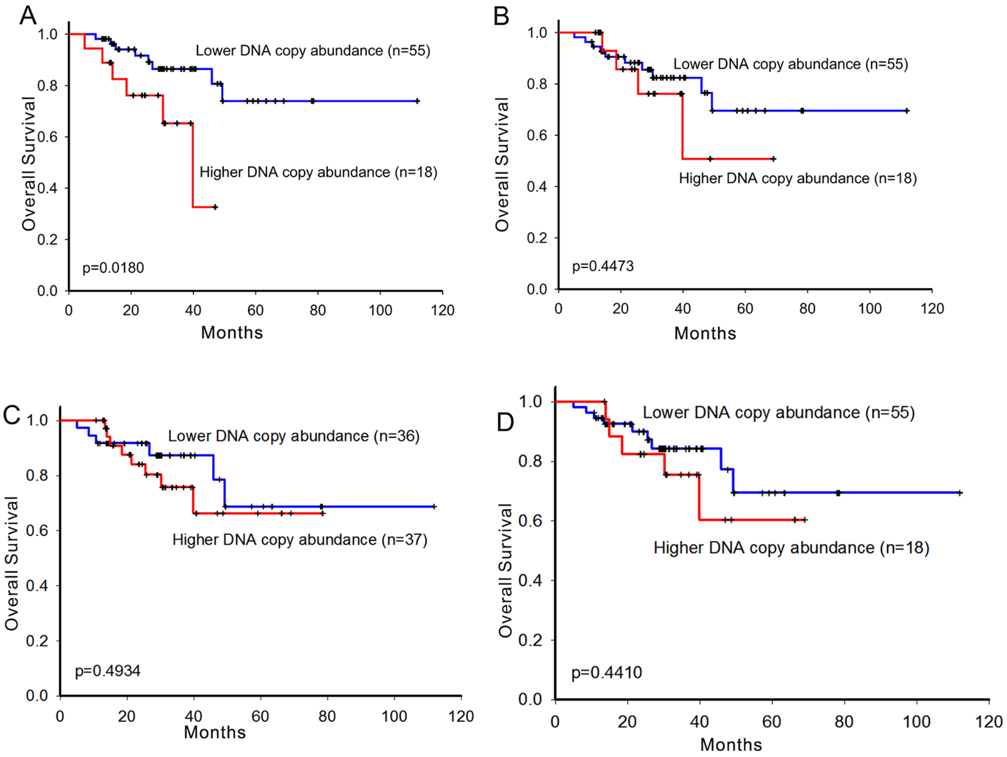

Supplementary Figure S2: Survival prediction by DNA copy number abundance of ErbB family in 73 patients carrying exon-19-deletion mutation. Kaplan-Meier curves for overall survival analysis on **A.** *EGFR*, **B.** *ERBB2*, **C.** *ERBB3*, and **D.** *ERBB4*. High- and low-risk groups are divided based on copy number abundance. The optimal cut points were determined by sensitivity analysis which provided the largest discrepancy in overall survival between the low- and high-risk groups on the basis of the log-rank statistic, respectively.

Supplementary Table S1: Best cut-off selection of genes for log-rank tests in *EGFR*

Percentage[¥]	cut-off[¥]	low-group[$]	high-group[$]	Log-rank test[#]	multi-cox[§]
20%	0.84604	52	209	0.6056	0.6951
25%	0.87978	65	196	0.2787	0.3353
30%	0.92348	79	182	0.1227	0.2584
35%	0.97266	92	169	0.1567	0.4363
40%	1.04582	105	156	0.441	0.7444
45%	1.10704	118	143	0.3482	0.4386
50%	1.16562	131	130	0.2015	0.2451
55%	1.22323	143	118	0.0962	0.1667
60%	1.30455	157	104	0.0776	0.2206
65%	1.37625	169	92	0.1082	0.2087
70%	1.51843	183	78	0.1157	0.0754
75%	1.60924	196	65	0.032	0.0113
80%	1.76262	208	53	0.0026	0.0016

[$] Number of samples in the low risk group and the high risk group respectively.
[#] P-value of log rank test between the low risk group and the high risk group.
[§] Multivariate Cox regression p-value
[¥] Cut-off percentage and CNA value of log-rank test'

Supplementary Table S2: Best cut-off selection of genes for log-rank tests in *ERBB2*

Percentage[¥]	cut-off[¥]	low-group[$]	high-group[$]	Log-rank test[#]	multi-cox[§]
20%	0.808647021	53	208	0.2151	0.2388
25%	0.846964849	66	195	0.2651	0.3439
30%	0.894690126	78	183	0.1401	0.2419
35%	0.923904731	92	169	0.291	0.4967
40%	0.961910786	105	156	0.0902	0.1695
45%	0.998804343	117	144	0.09	0.2347
50%	1.028109551	131	130	0.0231	0.1134
55%	1.079009076	143	118	0.0332	0.1834
60%	1.129359656	157	104	0.0708	0.2998
65%	1.178458537	169	92	0.0249	0.1097
70%	1.224950117	183	78	0.0315	0.1086
75%	1.279065371	195	66	0.006	0.0381
80%	1.346875967	208	53	0.015	0.0804

[$] Number of samples in the low risk group and the high risk group respectively.
[#] P-value of log rank test between the low risk group and the high risk group.
[§] Multivariate Cox regression p-value
[¥] Cut-off percentage and CNA value of log-rank test

Supplementary Table S3: Best cut-off selection of genes for log-rank tests in *ERBB3*

Percentage[¥]	cut-off[¥]	low-group[$]	high-group[$]	Log-rank test[#]	multi-cox[§]
20%	0.868364709	53	208	0.8696	0.4715
25%	0.906877757	66	195	0.2637	0.1571
30%	0.951590649	79	182	0.1571	0.1656
35%	0.997165587	92	169	0.1303	0.1659
40%	1.031623827	104	157	0.1138	0.1902
45%	1.068765246	118	143	0.0539	0.0631
50%	1.10013914	131	130	0.0202	0.0417
55%	1.169219666	144	117	0.1223	0.2609
60%	1.21460903	157	104	0.3307	0.664
65%	1.282811329	169	92	0.3546	0.8211
70%	1.386176533	182	79	0.2333	0.3629
75%	1.47698175	195	66	0.0351	0.1124
80%	1.632445695	208	53	0.0077	0.006

[$] Number of samples in the low risk group and the high risk group respectively.
[#] P-value of log rank test between the low risk group and the high risk group.
[§] Multivariate Cox regression p-value
[¥] Cut-off percentage and CNA value of log-rank test

Supplementary Table S4: Best cut-off selection of genes for log-rank tests in *ERBB4*

Percentage[¥]	cut-off[¥]	low-group[$]	high-group[$]	Log-rank test[#]	multi-cox[§]
20%	0.258800444	52	209	0.6266	0.3
25%	0.266846871	65	196	0.7379	0.4449
30%	0.28133618	79	182	0.7898	0.7115
35%	0.299292908	91	170	0.8291	0.9583
40%	0.308329963	105	156	0.6467	0.7646
45%	0.32122231	118	143	0.4409	0.5836
50%	0.340726958	131	130	0.2817	0.5307
55%	0.361801426	143	118	0.1146	0.2485
60%	0.376014838	156	105	0.0706	0.2198
65%	0.400672667	170	91	0.0419	0.1511
70%	0.432080791	182	79	0.0407	0.1309
75%	0.469248325	196	65	0.0163	0.0474
80%	0.535612673	209	52	0.093	0.2292

[$] Number of samples in the low risk group and the high risk group respectively.
[#] P-value of log rank test between the low risk group and the high risk group.
[§] Multivariate Cox regression p-value
[¥] Cut-off percentage and CNA value of log-rank test

Supplementary Table S5: Results of relapse-free survival analysis: multivariate cox regression in all patients and four sub-groups

Gene	Adjusted HR[$]	95% C.I.		*P* value[#]	Log-rank test	
	All patients (*n* = 261)				Cut-off[$]	*P*[§]
EGFR	1.14	0.76	1.71	0.543	75%	0.832
ERBB2	0.88	0.58	1.35	0.561	75%	0.924
ERBB3	1.09	0.76	1.55	0.651	50%	0.896
ERBB4	1.07	0.71	1.60	0.762	75%	0.949
	Wild type (*n* = 118)					
EGFR	0.79	0.39	1.61	0.515	75%	0.204
ERBB2	0.89	0.49	1.62	0.699	75%	0.739
ERBB3	0.97	0.60	1.59	0.907	50%	0.578
ERBB4	0.91	0.51	1.60	0.733	75%	0.613
	L858R (*n* = 70)					
EGFR	1.69	0.76	3.73	0.196	75%	0.171
ERBB2	1.02	0.41	2.51	0.970	75%	0.855
ERBB3	1.12	0.50	2.54	0.782	50%	0.832
ERBB4	0.98	0.41	2.39	0.972	75%	0.843
	Exon 19 deletion (*n* = 73)					
EGFR	2.72	1.12	6.59	0.027	75%	0.135
ERBB2	1.16	0.44	3.03	0.767	75%	0.676
ERBB3	1.58	0.68	3.68	0.284	50%	0.948
ERBB4	1.88	0.79	4.50	0.157	75%	0.634
	EGFR-activating mutations (*n* = 143)[+]					
EGFR	1.77	1.00	3.12	0.050	75%	0.040
ERBB2	0.91	0.49	1.71	0.776	75%	0.876
ERBB3	1.05	0.61	1.78	0.871	50%	0.807
ERBB4	1.22	0.68	2.19	0.511	75%	0.598

[#] Multivariate Cox regression *p*-value.
[§] P-value of log rank test between the low risk group and the high risk group.
[$] Cut-off percentage of log-rank test

Supplementary Table S6: Copy number abundance of ErbB family and correspondent clinical characteristics of each patient.

See Supplementary File 1
